# Bridging the Gap in Orofacial Pain Assessment for Individuals With Intellectual Disabilities: A Systematic Review of Validated Tools

**DOI:** 10.1111/scd.70097

**Published:** 2025-09-06

**Authors:** Bruna de Oliveira Rech, Ana Carolina Costa Borges, Jefferson R. Tenório, Beatriz Dulcineia Mendes Souza, Alessandra Rodrigues de Camargo

**Affiliations:** ^1^ School of Dentistry Federal University of Santa Catarina Florianópolis Brazil; ^2^ Department of Pathology and Oral Diagnosis Federal University of Rio de Janeiro Rio de Janeiro Brazil

**Keywords:** dental care for disabled, intellectual disability, pain measurement, systematic review

## Abstract

**Aims:**

Systematically review the literature to answer the focused question: “What is the best way to facilitate pain communication for patients with intellectual disabilities (ID) in dental care?”

**Methods:**

A systematic search strategy was conducted in five databases and gray literature. Studies evaluating pain communication in dental care for patients with ID were included. The risk of bias was assessed according to the Meta‐Analysis of Statistics Assessment and Review Instrument.

**Results:**

A total of 1525 studies were screened, and after applying exclusion criteria, 10 articles remained. The included studies were published between 2003 and 2021, with sample sizes ranging from 28 to 270 participants with developmental disabilities. The Dental Discomfort Questionnaire (DDQ) was the most commonly used tool in the studies. Most studies showed a low risk of bias.

**Conclusion:**

The DDQ is the most validated tool for assessing dental pain in individuals with ID, though evidence in adults is limited. Other tools provide useful behavioral cues but may not clearly differentiate pain from discomfort or anxiety. The absence of a gold standard underscores the need for context‐appropriate tool selection and clinician training to interpret nonverbal pain, enhancing diagnostic accuracy and promoting equitable care.

## 1 Introduction

Pain is defined as “an unpleasant sensation and an emotional experience associated with actual or potential tissue damage, or described in terms of such damage” [1]. Given its subjective and individual nature, self‐reporting is considered the gold standard for pain assessment [2]. However, this approach may not be suitable for individuals with significant cognitive impairments or limited verbal communication skills [3]. Individuals who cannot verbalize pain are often excluded from conventional diagnostic frameworks, increasing the risk of undetected distress and inadequate treatment. This underdiagnosis of pain is particularly concerning in populations with developmental disabilities (DDs), where communication limitations may limit the recognition of moderate‐to‐severe suffering [4].

DD refers to a broad group of severe chronic conditions that involve physical and/or intellectual impairments, including autism, Down syndrome, cerebral palsy, and intellectual disability (ID) [5]. Among these, ID, in particular, is characterized by deficits in intellectual functionating and adaptive behavior that emerge during the first 18 years of life [5]. ID affects an estimated 1% to 2% of the global population, with most individuals presenting mild‐to‐moderate impairments [6]. Despite this, many still face substantial barriers to expressing pain, especially in clinical settings where communication is central care. Consequently, painful conditions may be under‐recognized or misattributed to behavioral issues, leading to delays in diagnosis and treatment [7].

In the dental setting, painful processes can have various origins, with orofacial pains comprising a set of conditions, including pulp pain, periodontal pain, temporomandibular dysfunctions, neuropathies, neuralgias, and facial headaches [8]. The consequences of untreated odontogenic pain go beyond physical discomfort, contributing to reduced quality of life through sleep deprivation, emotional distress, reduced leisure, and dietary restrictions [9].

These manifestations are often misinterpreted or overlooked, especially in individuals with ID, who are more susceptible to experiencing higher levels of pain, have poorer oral health, and face significant challenges in verbally communicating their pain [8, 9]. Health professionals may attribute signs such as agitation, isolation, or aggression to the disability itself, rather than investigating them as possible expressions of pain [10, 11]. This phenomenon, known as diagnostic overshadowing, reinforces structural neglect and delays appropriate interventions. In more severe cases, undetected pain has been associated with self‐harm, functional regression, and unnecessary prescription of psychotropic medications [10, 11]. These outcomes reinforce the urgency of early and accurate detection through appropriate pain assessment strategies.

For diagnostic purposes, pain assessment can be performed using self‐report tools or observational behavioral analyses, which serve as valuable aids in clinical practice [2]. Although various observational instruments have been developed to measure pain in individuals with intellectual disabilities, there is no consensus on the optimal tool for this population [2]. Some scales are based on caregiver reports, others on facial expressions or body signals, but few have been validated specially for orofacial pain or systematically incorporated into dental care [2].

Despite advances in the development of these scales, no systematic study has yet comprehensively compiled and validated an instrument that could be considered the gold standard for pain assessment in individuals with ID. The lack of a standardized protocol generates diagnostic uncertainty and contributes to the persistent invisibility of this population's suffering in clinical settings. Furthermore, the absence of validated tools adapted to the dental context may perpetuate a cycle of missed diagnoses, delayed interventions, and worsening of oral health conditions. This gap in the literature highlights the need for a systematic review to synthesize existing evidence, identifies the most effective methods, and provides support for enhancing clinical practice and developing new assessment strategies.

Based on this context, the purpose of this systematic review is to answer the focused question: What are the most effective methods for communicating and assessing orofacial pain in dental office visits for patients with intellectual disabilities, considering the availability of validated and clinically adapted instruments for dental practice?

## 2 Methods

This systematic review was conducted based on the items from the Preferred Reporting Items for Systematic Reviews and Meta‐Analyses (PRISMA) checklist. The protocol for the systematic review was registered in the International Prospective Register of Systematic Reviews (PROSPERO) with the registration number **CRD42020187522**.

### 2.1 Eligibility Criteria

Studies were selected that evaluated pain communication in dental care for patients with ID, including individuals of all genders and without age restrictions. To reduce publication bias, studies without time and language constraints were included. Reviews, letters, personal opinions, book chapters, case reports, conference abstracts, animal research, and studies where patients had acquired intellectual disabilities (such as senile dementia)—or merely physical disabilities—were excluded.

### 2.2 Information Sources

The electronic search was conducted in the following databases: Latin American and Caribbean Health Sciences Literature (LILACS), PubMed/MEDLINE, Scopus, Cochrane, and Embase. Additionally, a search of the gray literature was conducted using Google Scholar, ProQuest, and Open Grey (Supporting Information). The Google Scholar search was limited to the first 100 published articles. Reference lists of the selected articles were also examined. The search across all databases, including gray literature, was performed on January 5, 2025.

### 2.3 Study Selection

The study selection was carried out in three phases. The first phase involved using reference management software (EndNote X7 Thomson Reuters, Philadelphia, PA, USA) to collect references and eliminate duplicates. In the second phase, A.C.C.B. and B.O.R. independently reviewed titles and abstracts of all the articles from the search. Articles that did not meet the inclusion criteria or met any of the exclusion criteria were excluded in this phase. In the event of uncertainty, A.R.C. was consulted. In the third phase, the remaining articles were read in full and assessed based on the same eligibility criteria.

### 2.4 Data Collection Process and Data Items

Initially, A.C.C.B. collected the necessary information from the selected studies. Subsequently, B.O.R. verified the integrity of the collected information. For each selected study, the following data were collected: authors, publication year, country, sample size, participants' age, main sample characteristics, pain grading tool used, main results, and conclusions.

### 2.5 Risk of Bias

A.C.C.B. and B.O.R. independently assessed the quality of the selected studies using the Meta‐Analysis of Statistics Assessment and Review Instrument (MAStARI). Any disagreements that arose were resolved with the assistance of A.R.C.

## 3 Results

### 3.1 Study Selection

**FIGURE 1:**
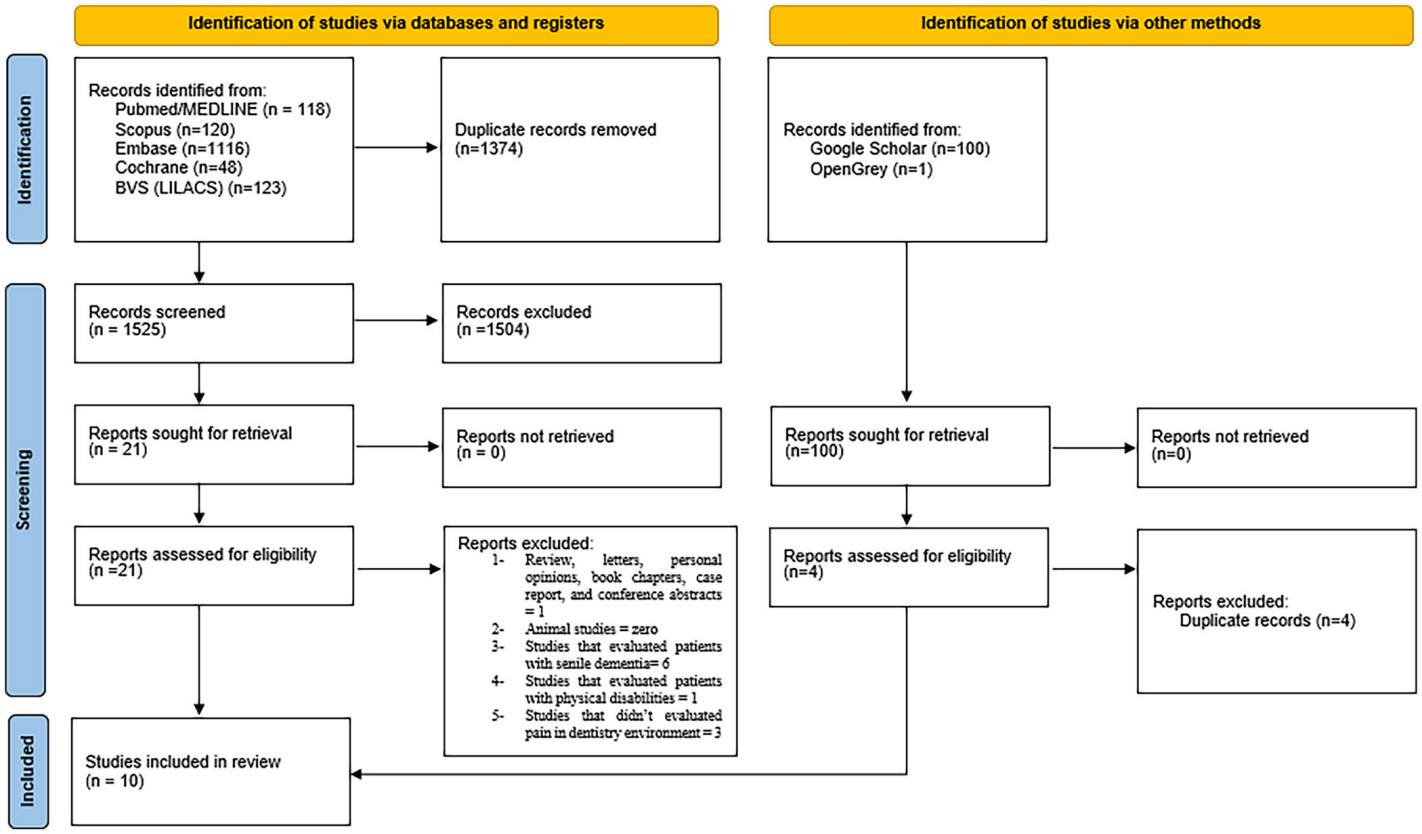
PRISMA 2020 Flow Diagram of the Study Selection Process.

During the initial search, 1525 citations were identified across the five electronic databases. After removing duplicate entries, 1374 remained. Following the review of titles and abstracts, 21 articles were deemed potentially useful and selected for evaluation in second phase. No studies from ProQuest, Open Grey, Google Scholar, or expert recommendations were added. Of the 21 works remaining for the third phase, 11 were subsequently excluded. Therefore, 10 articles were chosen for data collection to answer the research questions. Figure 1 illustrates the study identification and selection process.

### 3.2 Characteristics of the Studies

The search included articles published between 2003 and 2021 and encompassed studies conducted in eight different countries: Canada, the United States, Israel/Norway, Saudi Arabia, Switzerland, India, Turkey, and Belgium. The samples varied widely in size, ranging from 28 to 270 participants, and included individuals with physical disabilities and developmental disabilities, such as Down syndrome, cerebral palsy, and intellectual disability. A summary of the study characteristics can be found in Table 1.

**TABLE 1:** Summary of descriptive characteristics of included studies.

Year	Author	Country	Sample size (*N*)	Age (years)	Sample characteristics	Pain analysis tool	Main results	Conclusion
2003	Hennequin et al.	Canada	161	1–39	DS	OADS	Reports of pain experience for the DS and not non‐DS group were the same.	Parental perception of pain is less discriminant for children with DS than for their siblings without DS.
2005	Phan et al.	USA	28	40.61 (19–62)	MR	PADS	Scores on the PADS during the scaling procedure were greater than the scores of the other moments, but observations before and after scaling did not show statistical significance.	The authors conclude that the PADS may have utility in detecting pain and discomfort among patients with MR during medical and other procedures where a noxious stimulus is likely to be produced.
2008	Versloot et al.	Canada	56	7.5 (6–13)	Developmental Disability (DS, Cerebral Palsy, Autistic Traits)	DDQ	26% (*n* = 15) suffered from toothaches, as reported by the parents, and 33.3% of the parents (*n* = 11) were not sure if their child had a toothache, and no caries were found on examination.	The DDQ seems to be a functional and easy‐to‐use instrument for parents and caregivers to utilize in this group of children, to recognize if they have a toothache.
2010	Lotan et al.	Israel/Norway	59	42.1 (21–62)	ID	NCAPC	The mean NCAPC sum scores monitored across different situations showed significantly lower values (*p* < 0.05) at no‐pain situations (dormitory and dental clinic waiting room) than during pain situations (influenza injection and dental hygiene treatment).	In the present study, the ability of the NCAPC to distinguish between painful and non‐painful situations and between different painful interventions and the ability to capture pain at all levels of ID was indicated, suggesting that it is a valid scale for assessing acute procedural pain behaviors of adults, at different ages and at different levels of ID.
2012	Alaki et al.	Saudi Arabia	86	0–16	ID	DDQ	Caregivers of children with ID were more able to determine the severity of pain (mild, moderate, severe) with relation to the complexity of treatment than caregivers of healthy children (*P* = 0.003).	There is no significant difference in dental pain‐related behaviors among healthy and ID children. Caregivers of children with ID were more able to detect dental pain in their children compared to those of healthy children.
2015	Radha et al.	India	100	9–14	ID	DDQ	It was found that children with ID had a higher score of decayed and missing teeth, compared to NID controls. This difference was statistically significant. Children with ID also showed higher discomfort due to pain.	It was found that children with ID had a higher caries experience compared to no ID control and showed significantly higher pain experience than children with no ID.
2016	Krekmanova et al.	Sweden	270	12–18	Intellectual or Physical Disabilities	DDQ	Dental pain and discomfort, as measured by the total mean DDQ, was statistically significantly higher in the patients with disabilities when compared with the control group.	Children and adolescents with an intellectual or physical disability (D group) showed more pain and discomfort than the matched controls (C group), as measured by the Dental Discomfort Questionnaire (DDQ).
2017	Dugashvili et al.	Belgium	204	15–23	ID	UPAT	Results of the UPAT demonstrated functional TMJ pain in 32% of the athletes without significant prevalence. Joint sounds were found in 38% of subjects; 65% of these athletes also reported functional pain.	The UPAT demonstrated to be an additional tool to detect the existence of functional jaw pain possibly associated with TMD.
2019	Alzahrani et al.	Saudi Arabia	92	12–16	ID	DDQ	There was no statistically significant difference between mean DDQ scores in the ID group and in control participants who had caries.	There was a significant relationship between parents’ ability to determine dental pain and the complexity of dental treatment needed in both groups.
2020	Senirkentli et al.	Turkey	161	6–15	ID	DDQ	The DDQ scores of children with intellectual disabilities were mostly similar to those with normal cognition, except for higher scores in "pain when eating and brushing teeth".	The DDQ is an effective, functional, and user‐friendly tool for identifying dental pain in children with intellectual disabilities.

Abbreviations: DDQ, Dental Discomfort Questionnaire; DS, Down Syndrome; ID, Intellectual Disabilities; MR, Mental Retardation; NCAPC, Non‐Communicating Adult Pain Checklist; OADS, The Oral Assessment in Down Syndrome; PADS, Pain and Discomfort Scale; TMD, Temporomandibular Disorder; TMJ, Temporomandibular Joint; UPAT, Universal Pain Assessment Tool.

### 3.3 Risk of Bias in the Studies

Two of the 10 selected studies met all the methodological criteria of the MAStARI and, along with 7 others, were classified as having a low risk of bias. One study, however, was evaluated by the same instrument and deemed to have a moderate risk of bias. A detailed assessment of all aspects can be found in Table 2.

**TABLE 2:** Risk of bias assessed using the meta‐analysis of statistics assessment and review instrument (MAStARI) critical appraisal tools Risk of bias was categorized as high when the study reached up to 49% score “yes”, Moderate when the study reached 50% to 69% score “yes”, and Low when the study reached more than 70% score “yes”.

Question	Hennequin et al., 2003 [12].	Phan et al., 2005 [13].	Versloot et al., 2008 [14].	Lotan et al., 2010 [15].	Alaki et al., 2012 [16] ^.^	Radha et al., 2015 [17].	Krekmanova et al., 2016 [18].	Dugashvili et al., 2017 [19].	Alzahrani et al., 2019 [20].	Senirkentli et al., 2021 [21].
1. Is the study based on a random or pseudo‐random sample?	Y	Y	Y	Y	Y	Y	Y	Y	Y	Y
2. Are the criteria for inclusion in the sample clearly defined?	Y	Y	Y	Y	Y	Y	Y	N	Y	Y
3. Are confounding factors identified and strategies to deal with them stated?	Y	Y	Y	Y	Y	Y	Y	Y	Y	Y
4. Are outcomes assessed using objective criteria?	Y	Y	Y	Y	Y	Y	Y	Y	Y	Y
5. If comparisons are being made, is there sufficient description of groups?	Y	NA	Y	Y	Y	Y	Y	NA	Y	Y
6. Is follow‐up carried out over a sufficient time period?	NA	Y	Y	Y	Y	Y	Y	Y	Y	Y
7. Are the outcomes of people who withdraw described and included in the analysis?	Y	Y	Y	N	N	N	Y	N	N	N
8. Are outcomes measured in a reliable way?	Y	Y	Y	Y	Y	Y	Y	Y	Y	Y
9. Is an appropriate statistical analysis used?	Y	Y	Y	Y	Y	Y	Y	Y	Y	Y
%yes/risk	88.8%	88.8%	100%	88.8%	88.8%	88.8%	100%	66.6%	88.8%	88.8%

Abbreviations: N, no; NA, not applicable; U, unclear; Y, yes.

### 3.4 Individual Study Results

#### 3.4.1 Dental Discomfort Questionnaire

The Dental Discomfort Questionnaire (DDQ) was the most frequently used instrument, appearing in six studies assessing orofacial pain in children or adolescents with ID. In general, DDQ scores were higher among participants with dental caries or oral pain. Versloot et al. [12] modified the original scale, adding items such as excessive salivation and hand‐to‐mouth movements, aiming to increase the instrument's sensitivity and specificity. Krekmanova et al. [13] reported higher scores in children with severe ID compared to those with mild ID or physical disabilities. Alaki and Bakry [14] and Radha et al. [15] observed that caregivers were able to perceive signs of pain in children with ID, although in some cases DDQ scores did not show statistically significant differences between the groups. Alzahrani [16] and Senirkentli et al. [17] identified an association between higher DDQ scores and behaviors such as nighttime crying and difficulty brushing teeth, especially in participants with moderate‐to‐severe ID.

#### 3.4.2 Pain and Discomfort Scale

The Pain and Discomfort Scale (PADS) was used by Phan et al. [18] to detect pain and discomfort during ultrasonic scaling and oral prophylaxis in 28 individuals with ID and verbal communication difficulties. The results indicated that pain and discomfort levels were significantly higher during dental treatment. While PADS demonstrated sensitivity, it lacked specificity, making it difficult to distinguish between pain, anxiety, and general discomfort. Nevertheless, the authors considered it a suitable tool for detecting pain in this population.

#### 3.4.3 Non‐Communicating Adults Pain Checklist

The Non‐Communicating Children's Pain Checklist (NCAPC) was evaluated by Lotan et al. [19] for detecting pain before and during oral prophylaxis and vaccination procedures in 59 adults with varying levels of ID. Adapted from NCCPC, the NCAPC assesses six pain‐related behaviors: vocal reaction, emotional reaction, facial expression, body language, protective reaction, and physiological response. Scores were higher during dental procedures compared to the waiting room. The tool demonstrated both specificity and sensitivity across all levels of ID, effectively distinguishing painful from non‐painful situations, and was considered suitable for routine clinical use.

##### 3.4.3.1 Universal Pain Assessment Tool

The Universal Pain Assessment Tool (UPAT) was applied by Dugashvili et al. [20] to assess orofacial pain in 204 athletes with ID. Adapted from the Wong‐Baker FACES Pain Rating Scale, the tool incorporates facial illustrations and behavioral observations to identify pain in individuals with limited verbal communication. Clinical examinations evaluated mandibular movement and temporomandibular joint (TMJ) pain during both assisted and unassisted maximal mouth opening. Pain was assessed using the UPAT, with TMJ pain identified in 32% of athletes; among these, 74% were associated with temporomandibular disorder (TMD), generally described as mild. Pain was more frequently reported during mandibular movements, supporting the UPAT's utility in detecting pain in individuals with ID.

##### 3.4.3.2 Oral Assessment in Down Syndrome

One study by Hennequin et al. [21] employed the Oral Assessment in Down Syndrome (OADS) to evaluate 161 caregivers of children with Down syndrome and neurotypical children regarding oral health issues in these populations. Part of the questionnaire focused on caregivers’ perception of pain in the children, including difficulties in identifying its presence and location, as well as any painful oral conditions. The study found that the proportion of caregivers reporting difficulties in perceiving pain in children with Down syndrome remained consistent across all age groups, whereas this proportion decreased with age in the neurotypical group. These findings highlight the challenges caregivers face in interpreting pain and reinforce the value of structured tools for assessing nonverbal populations.

## 4 Discussion

The difficulty in defining symptoms of pain and subsequently forming a diagnostic process in individuals with ID primarily arises from communication issues, which are often neglected in clinical practice [22]. To address this problem, self‐report scales or observational methods have been employed based on behavioral indicators [3].

Self‐report scales are commonly used as the first option for pain assessment; however, their reliability is often questioned in populations with communication or cognitive limitations. In such cases, observational scales are considered “gold standard” [2, 3, 22, 23]. Some authors recommend combining observational and self‐report measures, even for individuals with neurotypical intellectual development [23]. However, most available instruments were developed for the pediatric population, and their validity in adults remains poorly established. Cognitive variation among ID makes the assessment process even more complex. Before choosing an assessment tool, it is essential to consider the individual's level of intellectual functioning and adaptative behavior in order to select the most appropriate strategy [2].

Cognitive variation within the ID population—ranging from mild to profound impairment—affects both pain expression and interpretation. Age is another critical factor: While most instruments have been developed and validated in pediatric populations, their utility in adults remains insufficiently established. This lack of age‐specific adaptation may lead to the under‐recognition of pain in adults with ID, particularly among older individuals who present additional comorbidities and atypical symptom expression.

Discomfort and pain play a significant role in triggering or exacerbating disruptive behaviors, which, in individuals with ID, may not only lead to self‐injury but also worsen stereotypical movement disorders [2]. These behaviors are frequently misunderstood and mistakenly attributed to the disability itself [24]. This phenomenon, known as diagnostic overshadowing, contributes to delayed diagnoses, inadequate treatments, and systemic neglect within health care systems. The medical literature highlights that communication limitations increase the risk of clinical errors and underdiagnoses in individuals with ID, with significant consequences for their health outcomes [10].

A classic example of this clinical bias is observed in emergency medicine: Shefer et al. [25] demonstrated that patients with mental disorders frequently have their physical symptoms misinterpreted as psychiatric manifestations, resulting in delays in both diagnosis and treatment. More recently, Dell'Armo and Tassé [26] reviewed 25 studies on diagnostic overshadowing and confirmed that this bias remains prevalent—particularly when health professionals lack specialized training to recognize medical conditions in individuals with intellectual disabilities. These findings underscore a structural pattern that likely extends to dentistry as well, where orofacial pain in individuals with intellectual disabilities may be erroneously attributed to the disability itself, rather than investigated as a legitimate clinical symptom.

Pain in this population is often expressed through subtle or atypical behaviors. Understanding the typical behavioral patterns of individuals with ID can aid in recognizing pain expression [3]. Establishing a pain history and behavioral profile is particularly helpful for identifying pain symptoms [3]. Individuals with ID often express pain and anxiety in ways that are not easily recognized [27]. The dental environment typically evokes fear and discomfort—and this is no different for this population [28]. Owing to associated oral health problems resulting from poor hygiene, craniofacial developmental disorders, and systemic medications [27], as well as infrequent dental visits, these patients are at increased risk of developing orofacial pain that is only identified when symptoms become severe [12]. To avoid inaccurate assessment and undertreatment, the use of observational tools is recommended in dentistry [27].

Numerous instruments exist for grading pain in individuals with cognitive impairment, yet few have been tested or developed to detect odontogenic pain [27]. This review found five different multidimensional instruments for assessing dental pain and/or discomfort in patients with ID. Of the 10 studies included, six used the DDQ scale for assessing orofacial pain [12, 13, 14, 15, 16, 17].

The DDQ was the most frequently used instrument and the best adapted to pediatric populations. Developed and validated by Versloot et al. [12], it assesses behaviors associated with odontogenic pain. The questionnaire is completed by parents or caregivers in two steps: first, evaluating the presence of dental pain, and then analyzing behaviors linked to painful oral processes. All six studies considered the DDQ to be an excellent tool—easy to use, with good validity and reliability—demonstrating significant clinical utility in detecting dental pain in children with ID [12, 13, 14, 15, 16, 17]. However, no study has yet confirmed its effectiveness in older adolescents or adults with ID, which limits its generalizability across the full spectrum of ages and cognitive profiles.

Findings from this tool revealed no significant differences in behaviors associated with dental pain between neurotypical individuals and those with ID, although children with disabilities demonstrated excessive salivation and more frequent hand‐to‐mouth actions [14]. Only one study correlated odontogenic pain to ID severity, showing that greater cognitive impairment increased the likelihood of pain and discomfort manifestation [13]. Overall, studies emphasized that children with ID experience more oral pain and discomfort than those without cognitive disorders [13, 15, 16].

Several instruments have been used in the dental context, including the OADS, PADS, NCAPC, and UPAT. While these tools show promise, they also have limitations. PADS, based on the NCCPC, assesses pain and discomfort in individuals with moderate‐to‐severe intellectual disabilities and communication difficulties, with good sensitivity but low specificity and challenges in distinguishing pain types. Despite this, Phan et al. [18] endorsed its dental use. Lotan et al. [19] adapted the NCCPC‐R for adults with ID, noting that prior pain experiences, age, and health status influence pain expression [29]. NCAPC, deemed suitable for acute pain assessment in young adults with ID by Breau and Burkitt [2], offers better sensitivity and specificity than PADS. OADS evaluates oral health issues and caregivers’ perception of orofacial pain in individuals with Down syndrome [21]. UPAT, derived from the Wong‐Baker FACES Pain Rating Scale, uses facial cues and behavior to assess pain when verbal communication is impaired and was applied by Dugashvili et al. [20] to detect orofacial pain in athletes with ID.

The scales analyzed based on pain‐associated behaviors, derived from various methods and specific purposes. From a broader perspective, these instruments evaluate facial expression, body activity, vocalizations (groans, cries, and screams), and sociability through various items to create a score that encodes pain presence or absence. Among all measures, the DDQ by Versloot et al. was the most studied in the dental context [12, 13, 14, 15, 16, 17]. However, despite their contributions, all these instruments rely on behavioral indicators that do not always fully capture the subjective nature of pain, especially in individuals with profound intellectual disabilities or concurrent physical impairments. The absence of a universal gold standard continues to generate diagnostic uncertainty.

This review presents some limitations that must be considered. The small number of included studies and the methodological heterogeneity—particularly regarding different tools, populations, validation methods, and study designs—may affect the comparability of findings. Most studies focused on children or adolescents with ID, leaving significant gaps in adult populations. Moreover, variability in participants’ cognitive profiles and clinical conditions may have influenced behavioral pain responses, complicating the generalization of results and making cross‐comparisons challenging. These gaps further underscore the need for age‐appropriate and cognitively adapted tools to improve diagnostic sensitivity in underrepresented subgroups, such as adults with severe or profound ID. Therefore, future studies should pursue more robust methodological standardization and validate scales specific to the dental context, aiming to enhance accuracy in detecting pain and discomfort in this vulnerable population.

Finally, although quantitative pain assessment remains the primary objective, analyzing the context of the pain experience is equally essential. Who is expressing pain, how it is expressed, and to whom, must all form part of the clinical response. Context‐sensitive tools, when combined with caregiver involvement and professional training, represent a necessary—though not sufficient—step. Employing such tools to identify odontogenic pain in individuals with ID and communication limitations enables the early recognition of pain‐related behaviors, helping to prevent undertreatment. In addition, it facilitates timely access to professionals capable of diagnosing and managing underlying conditions when pain is suspected, thereby contributing to improved quality of life. Without systemic change and inclusive health care strategies, the silent suffering of individuals with communication disabilities will remain invisible, perpetuating a cycle of clinical neglect and inequality.

## 5 Conclusion

Assessing pain in dental patients with ID requires effective tools due to verbal communication limitations and challenges in interpreting pain signals. Among the 10 studies reviewed, the DDQ was the most utilized tool adapted for clinical use in people with ID, while scales like the PADS and NCAPC showed utility in specific contexts and age groups, despite difficulties distinguishing pain from discomfort and anxiety. However, findings should be interpreted cautiously due to the small number of studies, heterogeneity in population (age, cognitive level, diagnosis), and inconsistent validation protocols, limiting generalizability across all ID subgroups. While DDQ shows promise in pediatric populations, its use in adults remains uncertain. Tools developed for medical or behavioral settings may require adaptation before routine dental application. Selecting tools suited to cognitive levels and training clinicians in nonverbal pain interpretation are crucial for accurate diagnosis. Continuous validation and integrated approaches could improve pain detection and treatment planning. Future research should focus on larger, methodologically rigorous studies stratified by cognitive profile and age, as well as direct comparisons of tool performance in real dental settings to establish clinical feasibility and accuracy. Until more robust evidence is available, using context‐sensitive tools alongside clinical judgement and caregiver involvement remains a pragmatic and ethical approach. Persisting without specific strategies risks normalizing the silent suffering of a historically overlooked population.

## Conflicts of Interest

The authors declare that they have no conflicts of interest.

## Supporting information




**Supplementary 1**. Search strategies
