# Supplementary material for: Bridging the Gap in Orofacial Pain Assessment for Individuals With Intellectual Disabilities: A Systematic Review of Validated Tools
Source: Spec Care Dentist. 2025 Sep 6;45(5):e70097. doi: 10.1111/scd.70097 (PMC12413780; doi:10.1111/scd.70097)
Supplement: Supplementary file 1 — Supplementary 1. Search strategies [file SCD-45-0-s001.docx]

**Supplementary 1 –Search strategies.**

| **Database** | **Search** (Jan 05, 2025) |
| --- | --- |
| **LILACS** | ("Intellectual Disability" OR "Intellectual Disabilities" OR "Intellectual Development Disorder" OR "Intellectual Development Disorders" OR "developmental disability" OR "developmental disabilities" OR "mental disability" OR "mental disabilities" OR "Mental Retardation" OR "Mental Retardations" OR "Mental Deficiencies" OR "Mental Deficiency" OR "Idiocy" OR "cognitive impairment" OR "cognitive impairments" OR "cognitive disability" OR "cognitive disabilities" OR "Dementia" OR "Mentally Disabled Persons" OR "disabled person" OR "disabled persons" OR "Mentally Disabled" OR "Dental Care for Disabled" OR "Deficiência Intelectual" OR "Transtorno do Desenvolvimento Intelectual" OR "Distúrbios do Desenvolvimento Intelectual" OR "Deficiência do Desenvolvimento" OR "Déficit intelectual" OR "Deficiência Mental" OR "Retardo mental" OR "comprometimento cognitivo" OR "deficiência cognitiva" OR "Demência" OR "Pessoas com Deficiência Mental" OR "Pessoa com deficiência" OR "Pessoas com deficiência" OR "Pessoas com Deficiências" OR "Assistência Odontológica para Pessoas com Deficiências" OR "Discapacidad intelectual" OR "Trastorno del desarrollo intelectual" OR "Alteración del desarrollo intelectual" OR "Discapacidad de desarrollo" OR "Déficit intelectual" OR "Discapacidad mental" OR "Retraso mental" OR "Discapacidad cognitiva" OR "Demencia" OR "Personas con discapacidad" OR "Persona con discapacidad" OR "Personas con discapacidades" OR "Atención Dental para Personas con Discapacidades") AND ("Pain Measurement" OR "Pain Measurements" OR "Pain Assessments" OR "Pain Assessment" OR "Analgesia Tests" OR "Analgesia Test" OR "Nociception Tests" OR "Nociception Test" OR "McGill Pain Questionnaire" OR "McGill Pain Scale" OR "Pain Scale" OR "Pain Scales" OR "Formalin Test" OR "Formalin Tests" OR "Pain Test" OR "Pain Tests" OR "pain perception" OR "pain perceptions" OR "pain questionnaire" OR "pain questionnaires" OR "pain evaluation" OR "pain evaluations" OR "sensory measurements" OR "sensory measurement" OR "Medição da dor "or "Avaliação da dor" OR "Teste de analgesia" OR "Testes de analgesia" OR "Testes de nocicepção" OR "Escala de dor" OR "Escalas de dor" OR "Questionário de dor de McGill" OR "Escala de dor de McGill" OR "Teste de dor" OR "Testes de dor" OR "percepção da dor" OR "questionário de dor" OR "avaliação da dor" OR "medidas sensoriais" OR "Questionários de dor" OR "Dimensión del Dolor" OR "Medición del dolor" OR "Evaluación del dolor" OR "Pruebas de analgesia" OR "Pruebas de nocicepción" OR "Escala de dolor" OR "Cuestionario de dolor de McGill" OR "Escala de dolor de McGill" OR "Prueba de dolor" OR "percepción del dolor" OR "Cuestionario de dolor" OR "Evaluación del dolor" OR "Mediciones sensoriales" OR "Cuestionarios de dolor") AND ("Dental Care" OR "dental" OR "dentistry" OR dentist* OR odontolog*) AND ( db:("LILACS")) |
| **PubMed/MEDLINE** | ("Intellectual Disability"[Mesh] OR "Intellectual Disability" OR "Intellectual Disabilities" OR "Intellectual Development Disorder" OR "Intellectual Development Disorders" OR "developmental disability" OR "developmental disabilities" OR "mental disability" OR "mental disabilities" OR "Mental Retardation" OR "Mental Retardations" OR "Mental Deficiencies" OR "Mental Deficiency" OR "Idiocy" OR "cognitive impairment" OR "cognitive impairments" OR "cognitive disability" OR "cognitive disabilities" OR "Dementia"[Mesh] OR "Dementia" OR "Mentally Disabled Persons"[Mesh] OR "disabled person" OR "disabled persons" OR "Mentally Disabled" OR "Dental Care for Disabled"[Mesh]) AND ("Pain Measurement"[Mesh] OR "Pain Measurement" OR "Pain Measurements" OR "Pain Assessments" OR "Pain Assessment" OR "Analgesia Tests" OR "Analgesia Test" OR "Nociception Tests" OR "Nociception Test" OR "McGill Pain Questionnaire" OR "McGill Pain Scale" OR "Pain Scale" OR "Pain Scales" OR "Formalin Test" OR "Formalin Tests" OR "Pain Test" OR "Pain Tests" OR "pain perception" OR "pain perceptions" OR "pain questionnaire" OR "pain questionnaires" OR "pain evaluation" OR "pain evaluations" OR "sensory measurements" OR "sensory measurement" OR (("Nonverbal Communication"[Mesh] OR "Nonverbal Communication" OR "Perception"[Mesh] OR "Perception" OR "Perceptions" OR "Surveys and Questionnaires"[Mesh] OR "Questionnaires" OR "Questionnairy") AND ("Pain"[Mesh] OR "Pain"[Title/Abstract] OR "Pains"[Title/Abstract]))) AND ("Dental Care"[Mesh] OR "dental"[Title/Abstract] OR "Dentistry"[Mesh] OR "dentistry"[Title/Abstract] OR "oral"[Title/Abstract] OR "Dentists"[Mesh] OR "Dentists" OR "Dentist") |
| **Scopus** | TITLE-ABS-KEY("Intellectual Disability" OR "Intellectual Disabilities" OR "Intellectual Development Disorder" OR "Intellectual Development Disorders" OR "developmental disability" OR "developmental disabilities" OR "mental disability" OR "mental disabilities" OR "Mental Retardation" OR "Mental Retardations " OR "Mental Deficiencies" OR "Mental Deficiency" OR "Idiocy" OR "cognitive impairment" OR "cognitive impairments" OR "cognitive disability" OR "cognitive disabilities" OR "Dementia" OR "disabled person" OR "disabled persons" OR "Mentally Disabled") AND TITLE-ABS-KEY("Pain Measurement" OR "Pain Measurements" OR "Pain Assessments" OR "Pain Assessment" OR "Analgesia Tests" OR "Analgesia Test" OR "Nociception Tests" OR "Nociception Test" OR "McGill Pain Questionnaire" OR "McGill Pain Scale" OR "Pain Scale" OR "Pain Scales" OR "Formalin Test" OR "Formalin Tests" OR "Pain Test" OR "Pain Tests" OR "pain perception" OR "pain perceptions" OR "pain questionnaire" OR "pain questionnaires" OR "pain evaluation" OR "pain evaluations" OR "sensory measurements" OR "sensory measurement" OR (("Nonverbal Communication" OR "Perception" OR "Perceptions" OR "Questionnaires" OR "Questionnairy") AND ("Pain" OR "Pains"))) AND TITLE-ABS-KEY("dental" OR "dentistry" OR "oral" OR "Dentists" OR "Dentist") |
| **Cochrane** | ("Intellectual Disability" OR "Intellectual Disabilities" OR "Intellectual Development Disorder" OR "Intellectual Development Disorders" OR "developmental disability" OR "developmental disabilities" OR "mental disability" OR "mental disabilities" OR "Mental Retardation" OR "Mental Retardations" OR "Mental Deficiencies" OR "Mental Deficiency" OR "Idiocy" OR "cognitive impairment" OR "cognitive impairments" OR "cognitive disability" OR "cognitive disabilities" OR "Dementia" OR "disabled person" OR "disabled persons" OR "Mentally Disabled") AND ("Pain Measurement" OR "Pain Measurements" OR "Pain Assessments" OR "Pain Assessment" OR "Analgesia Tests" OR "Analgesia Test" OR "Nociception Tests" OR "Nociception Test" OR "McGill Pain Questionnaire" OR "McGill Pain Scale" OR "Pain Scale" OR "Pain Scales" OR "Formalin Test" OR "Formalin Tests" OR "Pain Test" OR "Pain Tests" OR "pain perception" OR "pain perceptions" OR "pain questionnaire" OR "pain questionnaires" OR "pain evaluation" OR "pain evaluations" OR "sensory measurements" OR "sensory measurement" OR (("Nonverbal Communication" OR "Perception" OR "Perceptions" OR "Questionnaires" OR "Questionnairy") AND ("Pain" OR "Pains"))) AND ("dental" OR "dentistry" OR "oral" OR "Dentists" OR "Dentist") |
| **Embase** | ("Intellectual Disability" OR "Intellectual Disabilities" OR "Intellectual Development Disorder" OR "Intellectual Development Disorders" OR "developmental disability" OR "developmental disabilities" OR "mental disability" OR "mental disabilities" OR "Mental Retardation" OR "Mental Retardations " OR "Mental Deficiencies" OR "Mental Deficiency" OR "Idiocy" OR "cognitive impairment" OR "cognitive impairments" OR "cognitive disability" OR "cognitive disabilities" OR "Dementia" OR "disabled person" OR "disabled persons" OR "Mentally Disabled") AND ("Pain Measurement" OR "Pain Measurements" OR "Pain Assessments" OR "Pain Assessment" OR "Analgesia Tests" OR "Analgesia Test" OR "Nociception Tests" OR "Nociception Test" OR "McGill Pain Questionnaire" OR "McGill Pain Scale" OR "Pain Scale" OR "Pain Scales" OR "Formalin Test" OR "Formalin Tests" OR "Pain Test" OR "Pain Tests" OR "pain perception" OR "pain perceptions" OR "pain questionnaire" OR "pain questionnaires" OR "pain evaluation" OR "pain evaluations" OR "sensory measurements" OR "sensory measurement" OR (("Nonverbal Communication" OR "Perception" OR "Perceptions" OR "Questionnaires" OR "Questionnairy" ) AND ("Pain" OR "Pains"))) AND ("dental" OR "dentistry" OR "oral" OR "Dentists" OR "Dentist") |
| **Google Scholar** | ("Intellectual Disability" OR "Intellectual Disabilities" OR "Intellectual Development Disorder" OR "Intellectual Development Disorders" OR "developmental disability" OR "developmental disabilities" OR "mental disability" OR "mental disabilities" OR "Mental Retardation" OR "Mental Retardations" OR "Mental Deficiencies" OR "Mental Deficiency" OR "Idiocy" OR "cognitive impairment" OR "cognitive impairments" OR "cognitive disability" OR "cognitive disabilities" OR "Dementia" OR "disabled person" OR "disabled persons" OR "Mentally Disabled") AND ("Pain Measurement" OR "Pain Measurements" OR "Pain Assessments" OR "Pain Assessment" OR "Analgesia Tests" OR "Analgesia Test" OR "Nociception Tests" OR "Nociception Test" OR "McGill Pain Questionnaire" OR "McGill Pain Scale" OR "Pain Scale" OR "Pain Scales" OR "Formalin Test" OR "Formalin Tests" OR "Pain Test" OR "Pain Tests" OR "pain perception" OR "pain perceptions" OR "pain questionnaire" OR "pain questionnaires" OR "pain evaluation" OR "pain evaluations" OR "sensory measurements" OR "sensory measurement" OR (("Nonverbal Communication" OR "Perception" OR "Perceptions" OR "Questionnaires" OR "Questionnairy") AND ("Pain" OR "Pains"))) AND ("dental" OR "dentistry" OR "oral" OR "Dentists" OR "Dentist") |
| **OpenGrey** | ("Intellectual Disability" OR "Intellectual Disabilities" OR "Intellectual Development Disorder" OR "Intellectual Development Disorders" OR "developmental disability" OR "developmental disabilities" OR "mental disability" OR "mental disabilities" OR "Mental Retardation" OR "Mental Retardations" OR "Mental Deficiencies" OR "Mental Deficiency" OR "Idiocy" OR "cognitive impairment" OR "cognitive impairments" OR "cognitive disability" OR "cognitive disabilities" OR "Dementia" OR "disabled person" OR "disabled persons" OR "Mentally Disabled") AND ("Pain Measurement" OR "Pain Measurements" OR "Pain Assessments" OR "Pain Assessment" OR "Analgesia Tests" OR "Analgesia Test" OR "Nociception Tests" OR "Nociception Test" OR "McGill Pain Questionnaire" OR "McGill Pain Scale" OR "Pain Scale" OR "Pain Scales" OR "Formalin Test" OR "Formalin Tests" OR "Pain Test" OR "Pain Tests" OR "pain perception" OR "pain perceptions" OR "pain questionnaire" OR "pain questionnaires" OR "pain evaluation" OR "pain evaluations" OR "sensory measurements" OR "sensory measurement" OR (("Nonverbal Communication" OR "Perception" OR "Perceptions" OR "Questionnaires" OR "Questionnairy") AND ("Pain" OR "Pains"))) AND ("dental" OR "dentistry" OR "oral" OR "Dentists" OR "Dentist") |
